# Adult Ectopic Cervical Thymic Tissue in relation to a Parathyroid Adenoma and a Papillary Thyroid Carcinoma: A Report of Two Cases

**DOI:** 10.1155/2020/9127635

**Published:** 2020-03-11

**Authors:** Vijay Shrawan Nijhawan, Roopali Sehrawat, Neeti Goyal, Manish Gupta

**Affiliations:** ^1^Department of Pathology, Maharishi Markandeshwar Institute of Medical Sciences and Research, MMDU, Mullana, Ambala, Haryana, India; ^2^Maharishi Markandeshwar Institute of Medical Sciences and Research, MMDU, Mullana, Ambala, Haryana, India; ^3^Naval Hospital, Karwar, Karnataka, India; ^4^Department of Otolaryngology, Maharishi Markandeshwar Institute of Medical Sciences and Research, MMDU, Mullana, Ambala, Haryana, India

## Abstract

Ectopic cervical thymus is a rare congenital anomaly, which results because of the failure of complete descent of the thymus. They are incidental findings in the young and may disappear during the early years of life; however, they have rarely been described in adults. Some of them may undergo hyperplasia or neoplastic transformation and become visible or cause symptoms. We report two rare cases of incidentally detected cervical thymic tissue in adults. In one case, the thymic tissue was seen adjacent to a parathyroid adenoma of the left inferior parathyroid gland. In the other, it was seen adjacent to the left inferior parathyroid gland in a case of papillary thyroid carcinoma. In both these cases, the ectopic thymic tissue was diagnosed as a result of pathological examination, not clinically by macroscopic appearance during operation or radiological evaluation. The finding needs to be noted as these ectopic foci can occasionally give rise to hyperplasia and neoplasms of the thymus.

## 1. Introduction

Ectopic cervical thymic tissue (ECT) is a rare anomaly; it may be associated with the parathyroid and thyroid glands because of their embryologic relationship [1]. It is a rare cause of neck mass and is seldom considered in their differential diagnosis. This finding is mainly reported in adolescents and children and may disappear during the early years of life; however, they have rarely been described in adults. Ectopic thymic tissue is mostly discovered incidentally when tissue is being examined for some other pathology [1]. In a few cases, the tissue may undergo hyperplasia or neoplastic transformation and become visible or cause symptoms [1].

We report two rare cases of ECT in adults, one associated with left inferior parathyroid adenoma and the other with papillary thyroid carcinoma. In both the cases, the finding was incidental on histopathology examination, not clinically by macroscopic appearance during operation or radiological evaluation. The cases should remind clinicians of the infrequent occurrence of ectopic thymic tissue caused by maldescent during early embryologic development.

## 2. Case Reports

### 2.1. Case 1

A 42-year-old lady presented with a history of bony pains, multiple fractures, and weight gain over the last 4 years and was bed bound for the last 12 months. Imaging studies revealed severe osteopenia with multiple lytic skeletal lesions. Serum calcium was 12.4 mg/dL, and phosphorus was 2.8 mg/dL. Serum parathyroid hormone was elevated to 1058 pg/mL, and vitamin D3 level was low. The patient was euthyroid (T3-1.18 ng/mL, T4-8.34 *μ*g/dL, and TSH-3.85 mIU/mL). On ultrasonography, both the thyroid lobes were normal in size and architecture. There was a focal oval hypoechoic area in the left lobe measuring 6 × 7.8 mm with no vascularity or calcification. Parathyroid glands were not visualized. Few enlarged discrete nodes were seen in the left supraclavicular region with the largest measuring 1.1 cm in diameter. The nodes revealed a normal hilum. Otolaryngology examination was normal.

With a clinical diagnosis of primary hyperparathyroidism, 99 m TcSestamibi scanning was performed which showed abnormal tracer uptake inferior to the left lobe of the thyroid, consistent with left inferior parathyroid adenoma. No other parathyroid was visualized. Left inferior parathyroidectomy was performed.

**Figure 1:**
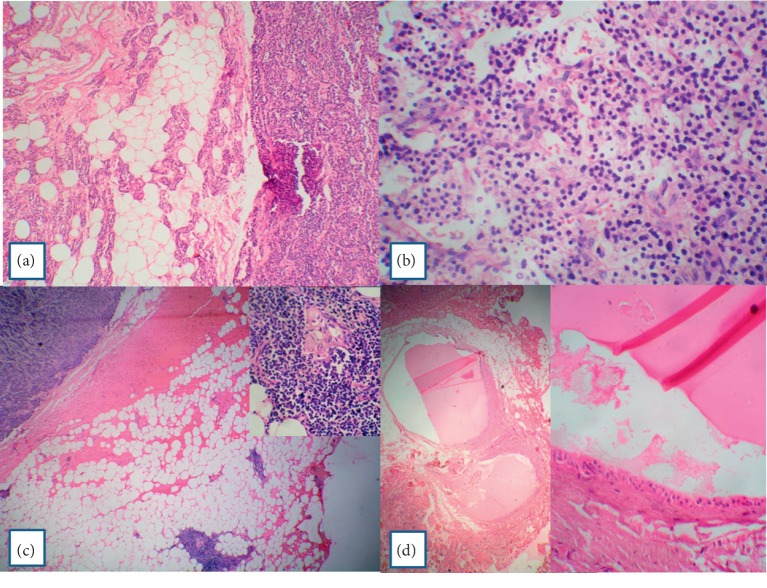
(a) Parathyroid adenoma with a rim of compressed normal parathyroid gland (H&E, ×100). (b) Parathyroid adenoma showing proliferation of chief cells with intervening blood vessels. No adipose tissue is seen (H&E, ×100). (c) Parathyroid adenoma with ectopic thymic tissue in the adjacent adipose tissue (H&E, ×100). Inset: higher magnification to show Hassel's corpuscle. (d) Parathyroid microcysts adjacent to the parathyroid adenoma (H&E, ×400). The cysts are lined by flattened to stratified lining epithelium (H&E, ×400).

Histopathology showed a parathyroid adenoma. Scant normal parathyroid tissue was seen compressed at one edge (Figure 1(a)). The adenoma was made up of chief cells within a delicate capillary network. Focal areas showed follicles and areas of hemorrhage (Figure 1(b)). The adjacent adipose tissue showed foci of ectopic thymic tissue comprising of lymphoid aggregates with interspersed Hassel's corpuscles (Figure 1(c)). A couple of parathyroid microcysts, around 2 mm in diameter and containing colloidal material, were seen in the surrounding adipose tissue. The cysts were lined by single to stratified cuboidal epithelium (Figure 1(d)).

Postoperatively, the patient was doing well clinically but was lost to follow-up after 6 months.

### 2.2. Case 2

A 30-year-old lady presented with midline neck swelling which moved with deglutition and was present since last 6 years. The patient was clinically and biochemically euthyroid (T3-0.86 ng/mL, T4-8.3 *μ*g/dL, and TSH-3.39 mIU/mL). On ultrasound, a solitary nodule 4 × 3 × 1 cm was seen in the left lobe of the thyroid. There was no significant cervical lymphadenopathy. Otolaryngology examination confirmed the presence of palpable left thyroid nodule, else was normal.

**Figure 2:**
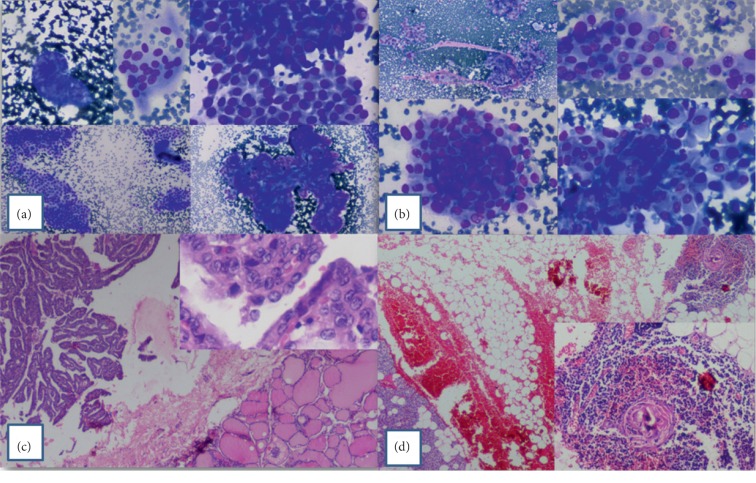
(a) FNA smears show papillae with and without vascular cores, multinucleate giant cells, monolayered sheets, and cells with well-defined cytoplasm (MGG). (b) FNA smears show chewing-gum colloid, intranuclear cytoplasmic inclusions, epithelial swirls, and cells with metaplastic cytoplasm (MGG). (c) Papillary thyroid carcinoma (classical) with thin arborizing papillae (H&E, ×40). Inset: enlarged oval, overlapping, hypochromatic nuclei. Pseudoinclusions and nuclear grooves are seen (H&E, ×400). (d) Inferior parathyroid gland with ectopic thymic tissue in the adjacent adipose tissue (H&E, ×100). Inset: higher magnification to show Hassel's corpuscle.

On fine-needle aspiration cytology, the case was diagnosed as papillary thyroid carcinoma. Cytology showed papillae with and without vascular core, monolayered sheets, and cells with metaplastic and well-defined cytoplasm. Epithelial swirls, chewing-gum colloid, and many cells with intranuclear cytoplasmic inclusions were noted (Figures 2(a) and 2(b)). The patient underwent near total thyroidectomy. The specimen measured 4 × 3.5 × 1 cm. There was a single encapsulated nodule (3.5 × 3.2 × 0.8 cm).

Histopathology confirmed the FNA diagnosis. Sections showed a tumor with arborizing papillae having delicate fibrovascular core. The tumor cells were polygonal with lightly eosinophilic cytoplasm. The nuclei were large, ovoid, and showed ground glass or hypochromatic appearance. Some showed nuclear grooves and pseudoinclusions (Figure 2(c)). In one of the sections, parathyroid tissue was seen, and in the adjacent adipose tissue, foci of ectopic thymic tissue comprising of Hassel's corpuscles in a lymphoid background was noted (Figure 2(d)).

The patient is on regular follow-up for the last 2 years. There has been no recurrence of the tumor.

## 3. Discussion

There is an intimate anatomical and embryological relationship between the inferior parathyroid gland and the thymus. Both are derived from the third pharyngeal pouch and descend down the neck to reach their anatomical position. Any maldescent can lead to ectopic thymic tissue or its lesion being encountered anywhere from the angle of the mandible to the superior mediastinum [1]. These are mostly encountered near the parathyroid as in our cases, but have also been described embedded within the thyroid [2–4], which at times has been mistaken on ultrasound examination as papillary thyroid carcinoma [4].

ECT has been commonly reported in the pediatric literature, mostly at autopsy, and uncommonly presents as a neck mass [1, 5, 6]. Most of the time, the finding may be incidental, correlating with a period of maximum growth of the thymus, but only rarely in adults, as they involute. Histologically, it may present as solid thymic tissue, as in our two cases, thymic cysts [6], but may also undergo hyperplasia [7] and transformation to thymoma [8]. In both the cases, the finding was incidental on histopathology examination, not clinically by macroscopic appearance during operation or radiological evaluation.

ECT has been described in the vicinity of a parathyroid adenoma [9, 10]. Ebrahimi et al [9] describe more than one ectopic tissue in the form of ectopic parathyroid tissue within ectopic thymic tissue. This has been ascribed to a close embryological relationship between the two. In Case 1, though no ectopic parathyroid tissue was noted, a couple of parathyroid microcysts were present in the vicinity of the parathyroid adenoma. Parathyroid (Kürsteiner's) cyst is an embryonal remnant of the duct connecting the parathyroid-thymus tissue in the III and IV pharyngeal pouches [11].

ECT has been peroperatively suspected to be a lymph node metastasis adjacent to a papillary thyroid carcinoma. Histopathology, however, revealed the nodule to be composed of ECT and parathyroid and thyroid tissue [12]. Case 2 presented in a similar manner; however, the parathyroid was not suspected to be a lymph node. Awareness of the ECT can avoid a morbid neck dissection.

## 4. Conclusions

Two rare cases of ectopic cervical thymic tissue in an adult adjacent to a parathyroid adenoma and a papillary thyroid carcinoma have been presented. Any ectopic tissue and their neoplasm such as thymic or parathyroid, even though very rare, should always be considered in the differential diagnosis when a neck mass is evaluated, especially in the pediatric age group. The cases should remind pathologists of the infrequent occurrence of ectopic thymic tissue caused by maldescent during early embryologic development.
